# Train-the-Trainers in hand hygiene: a standardized approach to guide education in infection prevention and control

**DOI:** 10.1186/s13756-019-0666-4

**Published:** 2019-12-30

**Authors:** Ermira Tartari, Carolina Fankhauser, Sarah Masson-Roy, Hilda Márquez-Villarreal, Inmaculada Fernández Moreno, Ma Luisa Rodriguez Navas, Odet Sarabia, Fernando Bellissimo-Rodrigues, Marcela Hernández-de Mezerville, Yew Fong Lee, Mohammad Hassan Aelami, Shaheen Mehtar, Américo Agostinho, Liberato Camilleri, Benedetta Allegranzi, Daniela Pires, Didier Pittet

**Affiliations:** 1 Infection Control Programme and WHO Collaborating Centre on Patient Safety, University of Geneva Hospitals and Faculty of Medicine, 4 Rue Gabrielle-Perret-Gentil, 1211 Geneva, Switzerland; 2 Institute of Global Health, Faculty of Medicine, University of Geneva, Geneva, Switzerland; 3 Faculty of Health Sciences, University of Malta, Msida, Malta; 4 Hotel-Dieu de Lévis, Lévis, Canada; 5 Department of Public Health, University of Guadalajara, Guadalajara, Jalisco Mexico; 6 Corporación Sanitaria Parc Taulí de Sabadell, Barcelona, Spain; 7 Hospital Universitario Principe de Asturias, Madrid, Spain; 8 Universidad Anáhuac, Naucalpan de Juárez, Mexico; 9 Department of Social Medicine, Ribeirão Preto Medical School, University of São Paulo, Ribeirão Preto, Brazil; 10 Hospital Nacional de Niños, de Costa Rica Dr. Carlos Sáenz Herrera, San José, Costa Rica; 11 Ministry of Health, Putrajaya, Malaysia; 12 Department of Pediatrics and Hand Hygiene and Infection Control Research Center, Imam Reza Hospital ,Mashhad University of Medical Sciences, Mashhad, Iran; 13 Infection Control Africa Network, Unit of IPC, Tygerberg Hospital, Cape Town, South Africa; 14 Department of Statistics and Operations Research, Faculty of Science, University of Malta, Msida, Malta; 15 Infection Prevention and Control Global Unit, Department of Service Delivery and Safety, World Health Organization, Geneva, Switzerland; 16 Department of Infectious Diseases, Centro Hospitalar Lisboa Norte and Faculdade de Medicina da Universidade de Lisboa, Lisbon, Portugal

**Keywords:** Hand hygiene, Infection prevention and control, Simulation training, Education, Healthcare-associated infection, Train-the-Trainers, World Health Organization, Improvement, Multimodal strategy, Implementation, Behavioural change

## Abstract

**Background:**

Harmonization in hand hygiene training for infection prevention and control (IPC) professionals is lacking. We describe a standardized approach to training, using a “Train-the-Trainers” (TTT) concept for IPC professionals and assess its impact on hand hygiene knowledge in six countries.

**Methods:**

We developed a three-day simulation-based TTT course based on the World Health Organization (WHO) Multimodal Hand Hygiene Improvement Strategy. To evaluate its impact, we have performed a pre-and post-course knowledge questionnaire. The Wilcoxon signed-rank test was used to compare the results before and after training.

**Results:**

Between June 2016 and January 2018 we conducted seven TTT courses in six countries: Iran, Malaysia, Mexico, South Africa, Spain and Thailand. A total of 305 IPC professionals completed the programme. Participants included nurses (*n* = 196; 64.2%), physicians (*n* = 53; 17.3%) and other health professionals (*n* = 56; 18.3%). In total, participants from more than 20 countries were trained. A significant (*p* < 0.05) improvement in knowledge between the pre- and post-TTT training phases was observed in all countries. Puebla (Mexico) had the highest improvement (22.3%; *p* < 0.001), followed by Malaysia (21.2%; p < 0.001), Jalisco (Mexico; 20.2%; p < 0.001), Thailand (18.8%; p < 0.001), South Africa (18.3%; p < 0.001), Iran (17.5%; p < 0.001) and Spain (9.7%; *p* = 0.047). Spain had the highest overall test scores, while Thailand had the lowest pre- and post-scores. Positive aspects reported included: unique learning environment, sharing experiences, hands-on practices on a secure environment and networking among IPC professionals. Sustainability was assessed through follow-up evaluations conducted in three original TTT course sites in Mexico (Jalisco and Puebla) and in Spain: improvement was sustained in the last follow-up phase when assessed 5 months, 1 year and 2 years after the first TTT course, respectively.

**Conclusions:**

The TTT in hand hygiene model proved to be effective in enhancing participant’s knowledge, sharing experiences and networking. IPC professionals can use this reference training method worldwide to further disseminate knowledge to other health care workers.

## Background

Healthcare-associated infections (HAIs) are associated with long-term morbidity, prolonged length of hospital stay, financial losses for hospitals and patients and higher mortality [1, 2]. Hand hygiene is a core infection prevention and control (IPC) strategy with a high impact for the prevention of HAIs and for limiting the spread of antimicrobial resistance [3, 4].

There is substantial heterogeneity in hand hygiene education among IPC professionals worldwide [5]. Countries face many challenges that prevent the participation of healthcare workers (HCWs) in educational programmes, including the lack of available trained professionals and financial constraints [6, 7].

There is a recognized global, unmet need for well-trained hand hygiene observers [8]. The standardization of observations is limited by the lack of formal core competencies and certification for IPC professionals. Insufficient training of hand hygiene observers results in interpersonal differences and over or underestimation of compliance rates, limiting comparability [8–10]. A recent systematic review found that there is a large heterogeneity in the methodology used in studies claiming to use the direct observation method. In fact, studies referring to this method used various observation schemes: the World Health Organization (WHO) tools (45%), own institutions’ tool (24%), a WHO-modified tool (21%) and a minority did not even mention the observation method used [9].

Education and training is recommended as a core component for effective IPC programmes by the WHO [10–12]. Simulation-based training of HCWs in a practical, bedside and hands-on approach has shown to improve hand hygiene compliance and lower HAIs [13–17].

To support countries with the capacity building for training IPC professionals, the Infection Prevention and Control programme and WHO Collaborating Centre on Patient Safety (IPC/WCC) at the University of Geneva Hospitals and Faculty of Medicine, Switzerland, developed a TTT course in hand hygiene based on the Geneva hand hygiene promotion strategy at the origin of the WHO multimodal strategy [4, 18]. The potentially positive impact of hand hygiene training on improved knowledge of IPC practitioners was first described in a pioneering TTT pilot event organised by the IPC/WCC in Brazil in 2015 [19]. This course was then modified to become more interactive following feedback from course participants and became the TTT model described in this paper.

Globally, capacity building of HCWs has the potential for enhancing networking and collaboration among professionals and for sustaining improvement. The TTT approach aims to reach larger audiences through subsequent training led by former course participants themselves. There is no prior publication that describes a formal course for training IPC practitioners in hand hygiene including training of auditors in hand hygiene compliance monitoring using direct observation methods.

We describe a standardized approach to hand hygiene training using a “Train-the-Trainers” (TTT) course for IPC professionals and evaluate its impact on participants knowledge in six countries.

## Methods

### Overview of the Train-the-Trainers course

The TTT course in hand hygiene was launched in 2016 as a standardized approach to hand hygiene training. It consisted in a 3-day, 25-h face-to-face course facilitated by IPC practitioners trained and validated in hand hygiene by IPC/WCC faculty members, and local IPC focal points in respective countries.

A detailed programme agenda is available as additional file [see Additional file 1]. The course materials are currently available in English and Spanish; a French version is under development. All course materials are made available for modification and adaptation by course participants to encourage the organization of subsequent TTT courses.

All participants were required to have a basic understanding of English in order to participate in the TTT. In addition, the course content was translated into the local language and simultaneous interpretation was used for non-native English speaking countries. Faculty members and local organizers were present at all times throughout the course to answer any questions related to the interpretation of the information.

The TTT model is based on the WHO Multimodal Hand Hygiene Improvement Strategy [20, 21]. It was structured around: key principles for best practices in hand hygiene, implementation of a multimodal promotion strategy, behavioural change, innovation and recent scientific evidence. The WHO’s evidence-based *My 5 Moments for Hand Hygiene* (2009) was used as a standardized methodology to monitor hand hygiene compliance [see Additional file 2] [3, 22] consistently and provide performance feedback. Participants who successfully completed the TTT in hand hygiene course and the final assessment were provided with a certificate of attendance.

### Course structure and organization

The course consists of didactic lectures, simulation-based training and experiential participatory activities. These activities include: 1) role plays with bedside practical sessions using a patient simulator; 2) clinical scenarios that help develop skills to synthesize and apply the information in real life; and 3) presentations from course participants about challenges they face in the implementation of hand hygiene-related activities in their own clinical settings. The hands-on training focuses on direct observation of hand hygiene compliance monitoring, using video reviewing of clinical scenarios and role-plays encouraging participation and feedback. Course materials that have been developed include videos (available at https://www.youtube.com/channel/UC-ymOg8cGHAZvUddrmG6UTQ) and case scenario-based simulations. Additonal videos are available to illustrate TTT organization and participation (available at www.CleanHandsSaveLives.org).

### Participants and settings

Country IPC leads requested the IPC/WCC to conduct TTT training in hand hygiene locally through their IPC organizations, supported by the Ministry of Health or other institutions.

The target audience for the TTT courses were personnel from departments that supervise IPC activities at the participating healthcare facilities (HCFs), including IPC, infectious diseases, hospital quality assurance department and hospital epidemiology departments. Hospital managers, ward nurse managers and other health professionals were also welcomed. Participants who attended a TTT course between June 2016 and January 2018 in Iran, Malaysia, Mexico, South Africa, Spain, and Thailand were included in the current study.

### Survey

We conducted a quasi-experimental study to evaluate the impact of the TTT training course based on a questionnaire [see Additional file 3], completed by course participants before (pre-training baseline survey) and after (post-training survey) the TTT courses in the six countries. In addition, follow-up measurements (5 months, 1 and 2 years after the TTT course) were conducted in Jalisco (Mexico), Puebla (Mexico) and Madrid (Spain), respectively. The main purpose of the questionnaire was to evaluate knowledge related to microbial transmission during healthcare delivery, key principles for best practices in hand hygiene and the WHO direct observation method (https://www.who.int/gpsc/5may/tools/en/).

All participants were assessed under examination conditions with no help provided on the responses. The pre- and post-tests were carried out at the same time and place, under supervision by course faculty members and participants were not allowed to discuss throughout the testing period. Furthermore, participants were not allowed to keep a copy of the test, so they could not share it with others (in the case of several TTT in the same country). Questionnaires were counted and sealed in envelopes once these were submitted.

The survey took one hour to complete and was based on previous tools proposed by WHO to evaluate hand hygiene knowledge for HCWs (https://www.who.int/gpsc/5may/tools/evaluation_feedback/en/) and on materials developed to validate hand hygiene observers for a European multicenter study [23]. It included 22 multiple-choice questions measuring: knowledge about HAIs and hand hygiene key principles (*n* = 5); the WHO methodology for hand hygiene monitoring (8); and the capacity of the participants to identify hand hygiene opportunities in clinical scenarios (9).

### Hand hygiene training for observers

The IPC/WCC in Geneva uses a rigorous structured and systematic approach to hand hygiene monitoring to reduce inter-rater differences between observers [18]. Based on this approach, the TTT course aimed for participants to acquire monitoring skills to ensure reliable and reproducible use of the WHO hand hygiene compliance auditing tool [24] so that compliance data could be consistently measured [25–27]. To achieve this objective, we developed observer training materials, including explanations of the “*My 5 Moments for Hand Hygiene*” [3, 22], and in-house videos with scenarios from the clinical environment where participants were required to recognise the correct moments and document these on the appropriate data collection form (https://www.who.int/gpsc/5may/tools/en/). Other WHO guidance documents and training videos were also used (https://www.who.int/infection-prevention/tools/hand-hygiene/training_education/en/).

### Statistical analysis

Each participant was assigned a unique identifier to assure confidentiality and to facilitate linking survey responses across the two assessments. Consent was implied when participants completed the questionnaire. All data were collected on paper forms and entered in data templates in Excel. We calculated questionnaire scores based on correct and incorrect answers. For each correct answer, a score of 1 was awarded; half correct answers were awarded 0.5. The maximum score was 20.

By considering solely the participants who answered both the pre- and post-test, the number of correct answers was computed for each participant both in the pre- and post-tests. Moreover, the percentage of correct answers for the whole group was computed by averaging across all participants for both tests. Descriptive statistics were used to evaluate the results. The Wilcoxon signed-rank test was used to compare average percentage scores before and after training. This non-parametric test was used because the score distributions were skewed.

## Results

**Fig. 1:**
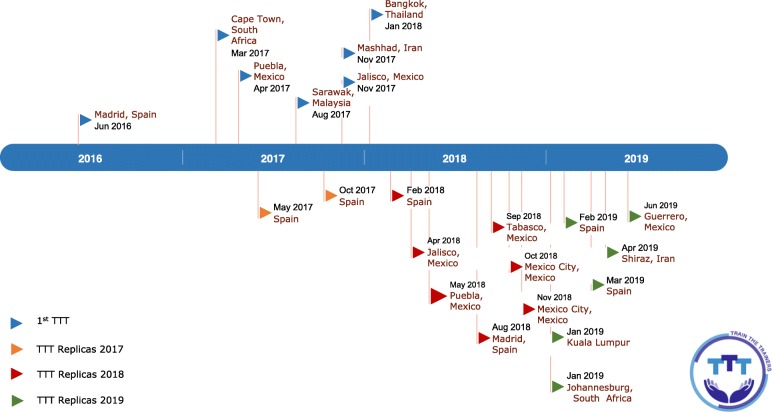
Train-the-Trainers in Hand Hygiene, June 2016–July 2019. Timeline chart showing the evolution of the Train-the-Trainers (TTT) programme between June 2016 and July 2019. *Replicas are organized by former TTT course participants and local IPC organizers. Note: The term TTT programme depicts the overall process that includes the original first courses and replicas

Between June 2016 and January 2018 the IPC/WCC held seven TTT courses in five middle-income countries (Iran, Malaysia, Mexico, South Africa and Thailand) and one high-income country (Spain). Fifteen TTT courses were then organized by previous course participants between 2017 and 2019 (Fig. 1). Here we describe the results from the initial seven courses (from June 2016 to Jan 2018).

A total of 305 IPC professionals completed the TTT programme. There was no withdrawal from the course. However, eight participants did not complete the pre-course questionnaire and six participants did not complete the post- course questionnaire due to logistical reasons (i.e. arriving late at the course venue or having to leave earlier). They were excluded from the analysis because we were not able to compare the participants’ knowledge acquisition before and after.

The average number of IPC professionals participating in a TTT course was 43, ranging from 21 to 81. Malaysia had the highest number of course attendees (*n* = 81) with participants from all 13 states and 2 federal territories in the country and 4 from Singapore. Thailand had participants (*n* = 53) from 11 different provinces; Jalisco (Mexico) had participants (*n* = 49) from 4 different cities within the State and Puebla (Mexico) had participants (*n* = 35) from 4 different States: Queretaro, Puebla, Jalisco and Mexico City. Iran had participants (*n* = 36) from 10 different cities in the country. South Africa had participants (*n* = 30) from 15 different (mostly English) speaking countries in Africa (Cameroon, Democratic Republic of Congo, Egypt, Ethiopia, Kenya, Liberia, Malawi, Namibia, Nigeria, Rwanda, Senegal, Sierra Leone, South Africa, Uganda, and Zimbabwe). Spain had participants (*n* = 21) coming mainly from Madrid and Barcelona. In total, participants from more than 20 countries were trained.

The majority of participants were nurses (*n* = 196; 64.2%), while physicians (n = 53; 17.3%) and other professionals (epidemiologists, quality assurance professionals and hospital managers (*n* = 56; 18.3%) were included.

The median duration of work experience in IPC participants was 2.8 years in Jalisco (Mexico), 3.5 in Malaysia, 4 in Thailand and Iran, 5 in Puebla (Mexico) and 10 in South Africa. This information was not available for Spain. All participants (*n* = 305) completed the questionnaire before and after the TTT courses held at all sites.

Most participants came from HCFs that engaged in the annual global WHO campaign ‘*SAVE LIVES: Clean Your Hands’* and celebrated the 5th of May World Hand Hygiene Day (https://www.who.int/gpsc/5may/registration_update/en/) (Spain 100%; South Africa 67%; Puebla (Mexico) 69%; Jalisco (Mexico) 75%; Malaysia 85%; Iran 73%; Thailand 33%).

The great majority of participants reported to have completed the Hand Hygiene Self-Assessment Framework (HHSAF)

(https://www.who.int/gpsc/country_work/hhsa_framework_October_2010.pdf): Spain 100%, South Africa 66.6%, Puebla (Mexico) 57.1%, Malaysia 85% and Iran 66.6%; but less frequently in Jalisco (Mexico) 44.9% and Thailand 45.2%.

**Fig. 2:**
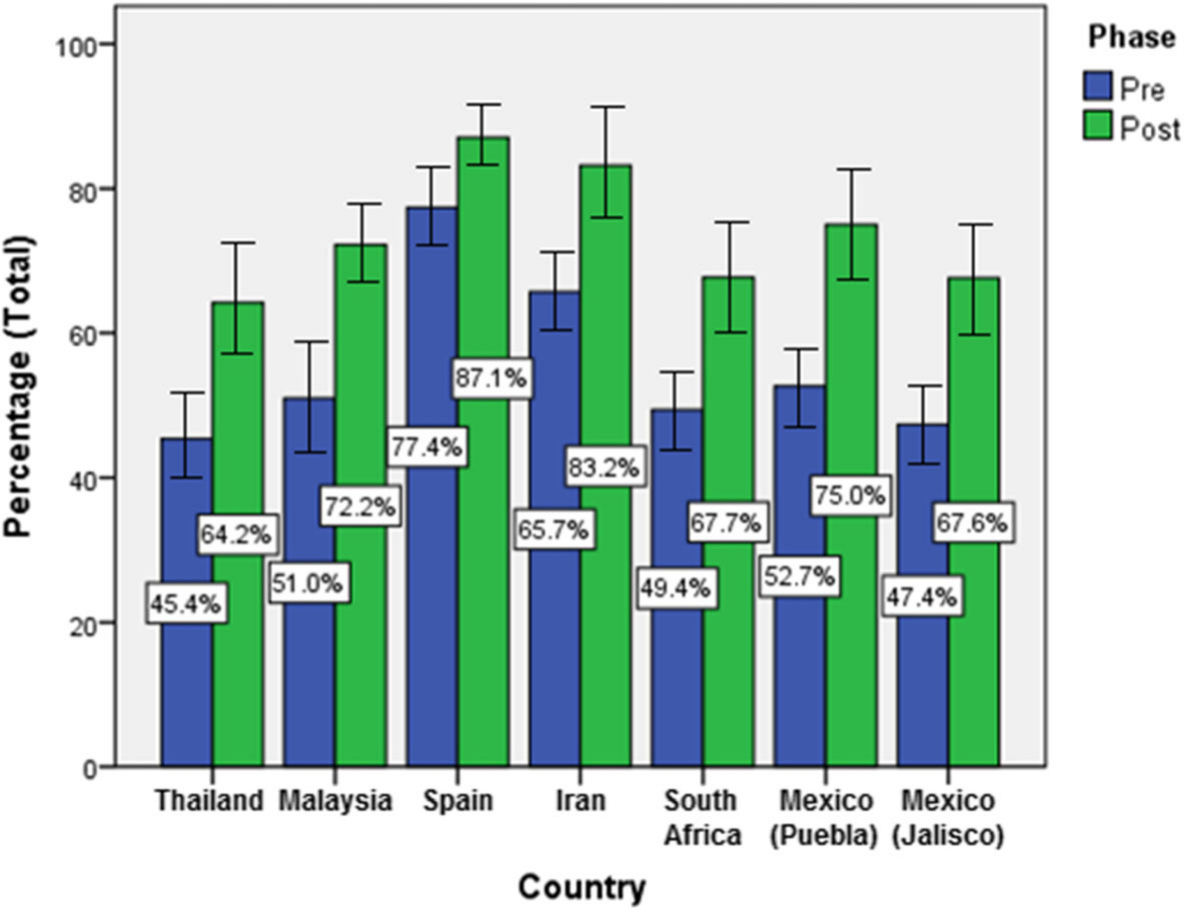
Train-The-Trainers: Improvement in Hand Hygiene Knowledge. Overall percentage of correct answers to the pre- and post-course questionnaire

Overall, we observed a significant improvement in knowledge of TTT programme participants across countries and regions (Fig. 2). This improvement was statistically significant (*P* < 0.05) in all countries between the pre- and post- phases (Table 1). Puebla (Mexico) had the largest improvement between the pre- and post-TTT course phases (22.3%). This was followed by Malaysia (21.2%), Mexico (Jalisco) (20.2%), Thailand (18.8%), South Africa (18.3%), Iran (17.5%) and Spain (9.7%). Spain had the highest pre- and post- overall scores. Thailand had the lowest pre- and post- overall scores.

**Table 1:** Improvement in Knowledge with Hand Hygiene among Train-The-Trainers Courses Participants, by Country and Regions

Country	Sample size	Percentage* (Pre)	Percentage* (Post)	z-score	*P*-value
Thailand	53	45.40%	64.20%	7.37	0
Malaysia	81	51.00%	72.20%	9.082	0
Spain	21	77.40%	87.10%	1.992	0.047
Iran	36	65.70%	83.20%	5.071	0
South Africa	30	49.40%	67.70%	4.367	0
Mexico (Puebla)	35	52.70%	75.00%	5.173	0
Mexico (Jalisco)	49	47.40%	67.60%	6.714	0

Percentage of correct answers: pre- and post- course, including all countries

### Knowledge about hand hygiene key principles

In all countries and all core questions, there was an improvement in the knowledge about microbial transmission during healthcare delivery, HAIs and key principles for best practices in hand hygiene between the pre- and post-TTT course phases (Table 2 and Table 3) [see Additional file 4]. Puebla (Mexico) had the largest improvement in the knowledge score (25.0%). This was followed by South Africa (22.8%), Jalisco (Mexico) (20.7%), Malaysia (16.9%), Thailand (15.2%), Iran (14.2%) and Spain (5.6%). The highest pre- and post- knowledge scores were recorded in Spain; the lowest in Malaysia.

**Table 2:** Knowledge about Hand Hygiene Key Principles: Pre- and Post-Course Evaluation among Train-The-Trainers Participants by Country and Regions

Country	Sample size	Percentage (Pre)	Percentage (Post)	z-score	P-value
Knowledge					
Thailand	53	62.70%	77.90%	3.059	0.002
Malaysia	81	48.40%	65.30%	4.344	0
Spain	21	84.50%	90.10%	1.07	0.285
Iran	36	72.90%	87.10%	2.638	0.008
South Africa	30	60.60%	83.40%	3.885	0
Mexico (Puebla)	35	54.40%	79.40%	3.806	0
Mexico (Jalisco)	49	49.50%	70.20%	4.226	0
Hand hygiene WHO methodology					
Thailand	53	52.50%	77.50%	4.688	0
Malaysia	81	61.80%	89.10%	5.635	0
Spain	21	71.90%	93.80%	2.07	0.038
Iran	36	76.90%	94.60%	2.884	0.004
South Africa	30	48.00%	69.70%	2.414	0.016
Mexico (Puebla)	35	65.70%	88.30%	3.22	0.001
Mexico (Jalisco)	49	55.90%	77.60%	3.186	0.001
Clinical Scenarios					
Thailand	53	33.80%	52.90%	5.114	0
Malaysia	81	43.90%	67.20%	5.083	0
Spain	21	61.90%	71.40%	0.655	0.516
Iran	36	44.60%	65.10%	2.744	0.006
South Africa	30	23.30%	30.80%	0.826	0.407
Mexico (Puebla)	35	37.10%	55.00%	2.203	0.028
Mexico (Jalisco)	49	25.20%	51.40%	4.676	0

Footnote: Pre- and post-course percentages of correct answers on three sections of the questionnaire 1) Knowledge about microbial transmission, healthcare-associated infections and key principles for hand hygiene best practices, 2) WHO methodology for hand hygiene observation, 3) clinical scenarios based on *My 5 Moments for Hand Hygiene*

**Table 3:** Core Questions from the course Questionnaire: Pre- and Post-Course Evaluation among Train-The-Trainers Participants

Question	Percentage (Pre)	Percentage (Post)	z-score	P-value
Knowledge				
Healthcare-associated infections	64.30%	78.70%	3.946	0
Microbial transmission	49.10%	65.90%	2.501	0.012
Hand rubbing/ hand washing	41.60%	71.10%	7.348	0
Alcohol-based hand rub	50.20%	74.30%	5.546	0
Glove use	69.20%	80.30%	3.169	0.002
WHO methodology				
Multimodal improvement strategy	44.90%	86.60%	10.835	0
Hand hygiene indications	62.30%	82.50%	2.397	0.016
Hand hygiene opportunities	49.50%	58.90%	2.737	0.006
The patient zone	75.70%	83.50%	2.29	0.022
Hand hygiene actions	66.70%	72.60%	1.351	0.177
Before clean/aseptic procedures	52.60%	76.70%	5.625	0
Clinical Scenarios				
Before touching a patient	20.00%	52.80%	8.415	0
Before clean/aseptic procedures	5.60%	28.30%	3.104	0.002
Before/after touching a patient	64.20%	90.60%	3.249	0.001
After/Before touching a patient	58.50%	84.90%	3.019	0.003
After touching a patient	32.10%	67.90%	3.691	0
Coincidence of indications	5.70%	32.10%	3.476	0.001
Peripheral venous catheter	38.70%	49.30%	2.536	0.011
Urinary catheter	49.30%	62.60%	2.791	0.005
After touching patient surroundings	46.50%	61.60%	3.62	0

Percentage of correct answers: pre- and post-course, including all countries and participants

### WHO methodology for hand hygiene observation

In all countries, there was an improvement in the score between the pre- and post-phases following the training on the WHO *My 5 Moments for Hand Hygiene* methodology (Table 3). This improvement was significant (*P* < 0.05) in all countries (Table 2), whilst Malaysia had the largest improvement (27.3%). This was followed by Thailand (25.0%), Mexico (Puebla) (22.6%), Spain (21.9%), South Africa (21.7%), Mexico (Jalisco) (21.7%), and Iran (17.7%) (Table 2) [see Additional file 4]. Iran had the highest pre- and post-hand hygiene scores on hand hygiene observation questions, whilst South Africa had the lowest scores.

### Clinical scenarios

In all countries, we observed an improvement between the pre- and post-phases in recognizing and identifying the *My 5 Moments for Hand Hygiene* from the clinical simulated scenarios provided in the questionnaire (Table 2 and Table 3). The improvement did not reach statistical significance in Spain and in South Africa. Jalisco (Mexico) had the largest improvement (26.2%), followed by Malaysia (23.3%), Iran (20.5%), Thailand (19.1%), Mexico (Puebla) (17.9%), Spain (9.5%) and South Africa (7.5%) [see Additional file 4]. Spain had the highest pre- and post- scores recorded, while South Africa had the lowest pre- and post-scores related to clinical scenario type questions.

### Sustainability in hand hygiene knowledge improvement

**Fig. 3:**
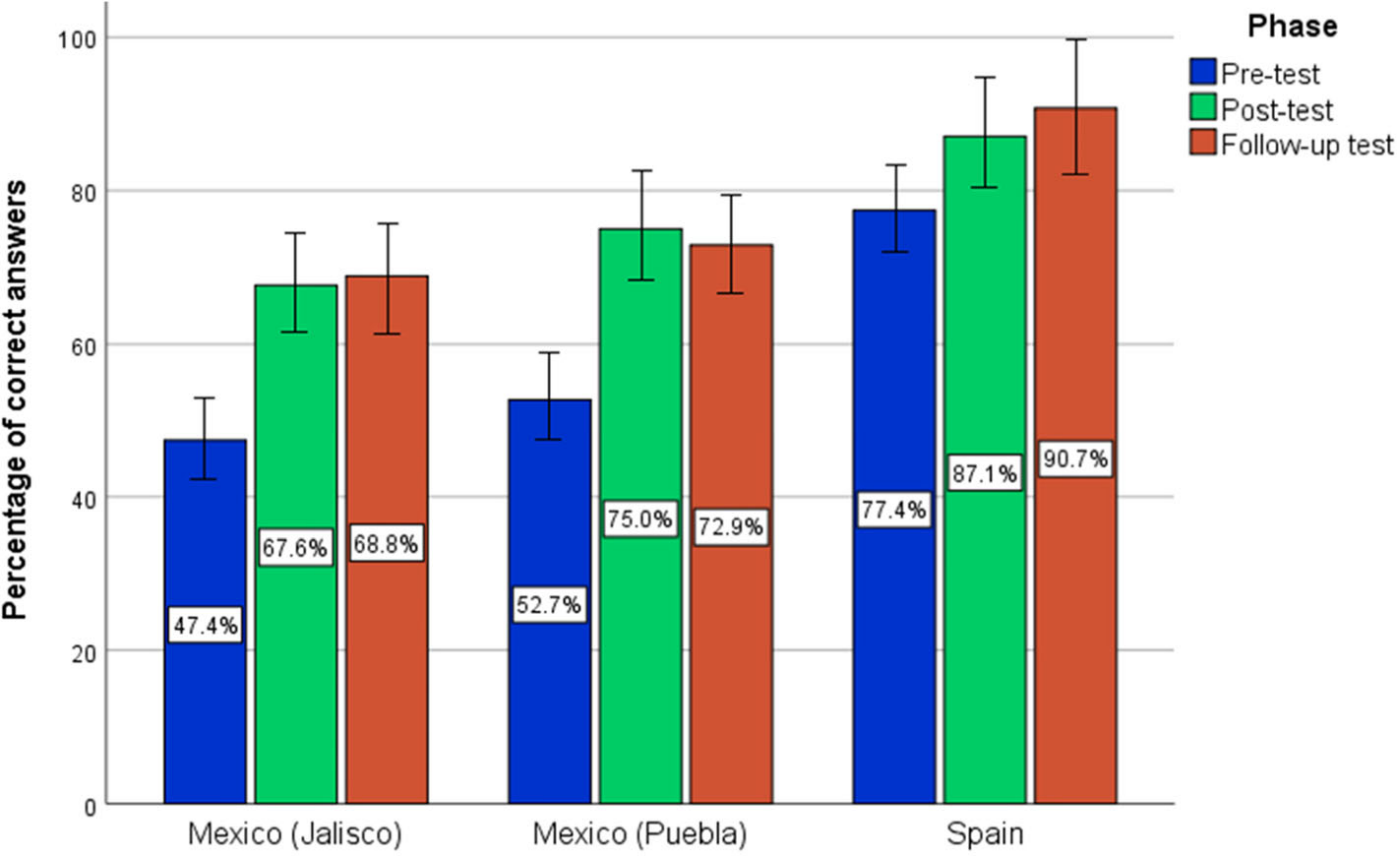
Sustainability of Hand Hygiene Knowledge following Train-The-Trainers Courses Overall percentage (mean + 95% CI) of correct answers to the pre-, post- and follow-up test in three sites (Jalisco, Puebla and Madrid 5 months, 1 year and 2 years after the first Train-The-Trainers course, respectively) between June 2017 and August 2018

Follow-up questionnaires were applied and analyzed in three original TTT courses sites in Jalisco (Mexico), Puebla (Mexico) and Madrid (Spain; Fig. 1). The average knowledge score increased from the pre-training baseline to the post-training phase (Fig. 3) and improvement was sustained in the last follow-up phase when recorded 5 months, 1 year and 2 years after the first TTT course in Jalisco, Puebla and Madrid, respectively (Fig. 3). Spain had the highest pre-, post- and follow-up overall scores as shown in the final measurement 2 years after the first TTT; ie, mean knowledge score 78.08.

### Case studies of success following the first train-the-trainers courses in Spain and Mexico

Spain and Mexico stood out as case studies of success in rolling out subsequent training programmes following the initial TTT. The PCI/WCC translated the course materials in Spanish. It was adapted to local needs with the preferred tools being: role plays, case scenarios based on their own clinical practice experience and the *My 5 Moments for Hand Hygiene* interactive material.

In Spain, six TTT replicas have been organized with the participation of former trainees since 2017. Courses are held twice per year, involving small groups of not more than 20 participants per course.

In Mexico five training workshops have been organized by former TTT certified course participants between 2018 and 2019; they were held in Jalisco (1), Tabasco (1), Mexico City (2), and Guerrero (1). A total of 278 course participants were trained; participants were personnel from the epidemiology department, IPC and quality assurance departments of HCFs with representatives from all Public Health Institutions and National Institutes.

A key physician and TTT course participant, now spearheading IPC initiatives in Mexico shared: *“Above all what I see is that those who attended the TTT course really changed their culture about hand hygiene, they no longer have to be told to do something, instead they have their own initiative”.*

Other positive aspects reported following TTT courses is the celebration of 5th of May (World Hand Hygiene Day), and many other initiatives to promote improved hand hygiene practices through organised events such as poster contests, the use of videos Ultra Violet hand hygiene education boxes, etc. Almost all participants commented that they had not received such intensive training on hand hygiene compliance monitoring and promotion before attending the TTT.

## Discussion

The launch of a hand hygiene simulation-based educational programme resulted in a significant increase in participants knowledge for all HCWs in a range of countries worldwide as measured by a pre-post questionnaire.

This is the first report on a worldwide TTT programme on key principles for best practices in hand hygiene showing a positive effect on knowledge. Despite the diverse context of languages, healthcare systems, educational background, resources and cultures, our study demonstrates the feasibility of a TTT educational model based on the WHO Multimodal Hand Hygiene Improvement Strategy [23] in different healthcare settings around the world. Our results expand and strengthen the findings from the first pioneering TTT event organised by the IPC/WCC in Brazil in 2015 [19]. Bellissimo-Rodrigues et al. [19] reported that an intensive 3-day TTT course in hand hygiene increased the knowledge of IPC professionals (*n* = 33) from 77.0% in the pre- to 89.7% in the post-training phase (*P* < 0.001). They have made available all the video recorded presentations in Portuguese and in English and these received a lot of attention (https://brasil.aesculap-academy.com/). The successful results from the first TTT in Brazil encouraged the replication of 25 hand hygiene courses with more than 800 trained IPC professionals nationwide.

The course methodology (i.e. simulation, scenario-based, bedside, hands-on training and individual experiences) showed improvement and the adaptability of the programme to the local context according to levels of progress, resources available and local culture. Simulation-based education has been proven to be an effective form of learning which leads to improved and lasting results [28–30] and is associated with decreased HAI rates and increased hand hygiene compliance [13–17]. The TTT promotes an interactive learning environment in small groups and encourages the application and synthesis of knowledge within a clinical context.

The benefit of the TTT programme was attested by improved in post-test scores. Our data show that overall the TTT course on hand hygiene best practices, WHO methodology for hand hygiene observations and clinical scenarios to assess *My 5 Moments for Hand Hygiene* was associated with significant improvement across all sites. The highest overall pre- and post-test scores were recorded in Spain and followed by Iran, while Thailand had the lowest scores. Spain is the only high-income country and this may possibly influence the high scores. We attributed the higher scores of IPC professionals in Spain and Iran to more appropriate IPC training and higher level of education before IPC specialisation [6, 7].

Hospital Infection Committees have been a legal obligation for all hospitals in Spain since 1987 [31] and a national hand hygiene programme has been promoted by the Ministry of Health Social Services and Equality since 2008 [32]. The Iranian Nosocomial Infection Surveillance System (INIS) was established in 2007 [33]. The findings of high scores from Iranian participants confirm the previously described acceptable knowledge and attitude scores of HCWs in Iran about hand hygiene [34]. On the other hand, in Thailand, the point prevalence of HAIs has been estimated at 6.5% with, on average, 250,000 patients affected each year [35]. In contrast, hand hygiene compliance rates of < 10% have been reported in the country [36, 37], clearly indicating that improvement of HCWs knowledge about HAIs and hand hygiene is needed. Our findings will stimulate the launch of coordinated educational activities that ultimately impact on improved hand hygiene compliance in countries worldwide.

Overall, the achievement of a greater improvement following the training programme was recorded in Mexico and Malaysia while greater knowledge improvement was achieved in Mexico and South Africa. Knowledge of course participants improved significantly after the TTT interventions across all sites, also it did not reach statistical significance between the pre- and post-test phases in Spain. Similarly, Allegranzi et al. [21] reported substantial progress and improvement with hand hygiene in low-and-middle-income countries (LMICs) than in high-income countries. In HCFs where basic IPC education and hand hygiene resources are scarce, the contribution of training could lead to immediate progress [21]. In addition, there is a need to increase training opportunities in IPC to expand the spread of educational training programmes to reach larger audiences including resource-limited settings. Training on hand hygiene is a priority across LMICs, where the educational needs of IPC professionals are not being met as they are assigned to the role with no previous training.

In Malaysia, the Ministry of Health (MoH), has set improved hand hygiene best practices as one of the priorities. Since 2013, the MoH has mandated all public HCFs to complete the WHO Hand Hygiene Self-Assessment Framework (HHSAF), a key tool aimed at tracking the level of progress of HCFs to improve best practices [38]. Following the TTT hand hygiene training in Malaysia, the IPC/WCC has been officially approved as a Continous Professional Development provider for all physicians in Malaysia to support with specialised education. Not all countries have the resources to organize local hand hygiene training modules: LMICs, in particular, tend to rely on international organizations to provide education and training. Training in hand hygiene multimodal improvement strategy should become part of IPC education and training certificates worldwide, as is the case already with the “European Certificate for Infection Control” established by the European Society for Clinical Microbiology and Infectious Diseases [5, 12, 13]. In addition, hand hygiene training should be part of the curricula of health sciences undergraduate courses.

Our data show that less than half of the participants across all countries, apart from Spain, were able to recognize correctly all *My 5 Moments for Hand Hygiene* in the practical scenario-based type of questions in the pre-test survey, regardless of the years of experience in IPC (see Additional file 4, figure on case scenario technical type questions to identify *My 5 Moment for Hand Hygiene*). Our findings confirm previous studies documenting low knowledge and adherence to the WHO 5 Moments for Hand Hygiene [21, 28]. The widespread nature of the challenges with hand hygiene direct monitoring and recognizing the correct 5 Moments highlights the need for standardized approaches to training IPC professionals. A recent multicenter study on hand hygiene improvement reported that Moment 2 before aseptic procedure and Moment 5 after touching patient’s surroundings were mentioned the least by study participants [29].

A structured and systematic approach to observation that entails auditor’s expertise and preparedness would be more rigorous at aiming to capture all Moments during an audit [9, 35, 36]. The hand hygiene programme in Geneva [39], an institution with a long history of hand hygiene promotion, [20, 32] uses experienced and validated hand hygiene observers to monitor compliance. Similarly, Hand Hygiene Australia requires hand hygiene auditors to attend auditor training, be validated and demonstrate related competencies to ensure standardization of auditors [40, 41].

Despite the proven success of the TTT programme, our study has limitations. First, the study had an observational design and course participants were selected from local organizers based on various reasons with no specific inclusion criteria. Therefore, selection bias may have reduced the generalizability of the results. Second, this was a quasi-experimental design; there was no control group and only pre- and post-measures were available. Furthermore, when assessing sustainability, other events in time may have confounded the results. However, the TTT serves as a guide for future interventions and as a starting point for other educational IPC interventions. Hand hygiene compliance before and after attending a TTT course was not monitored, and thus it remains unknown whether these events were associated with improved compliance. Subsequently, the association between improved knowledge scores after attending a TTT and HAI rates was out of the scope of the current study. Noteworthy, the pre-post and follow-up measures were based on a self-reported questionnaire tool. This limitation was minimised by ensuring that repeated measurements (follow-up phase) were carried out. Finally, the TTT requires professionals to take at least 3 days from work, and, in hospitals with limited human resources, attendance may be cumbersome and TTT requires some financial resources to be available.

Of future research interest is the evaluation of the impact of TTT training to improve hand hygiene compliance and best practices among HCWs and ultimately to reduce HAIs.

## Conclusions

In conclusion, the Geneva TTT hand hygiene programme is a unique, comprehensive programme that can be used and adapted in an international scale for improvements in hand hygiene knowledge and best practices (the reader can access video reports from TTT courses, see [42]). Our findings suggest that the TTT is a successful method for expanding the reach of standardized hand hygiene training packages at local and national levels. This approach provides a blueprint in countries where hand hygiene training is still absent; it also allows to improve knowledge in countries were national promotion strategies are in place. Following the initial TTT courses in the aforementioned countries, a number of countries replicated TTT events with former course trainees acting as instructors for consecutive courses. To date, Brasil, Iran, Malaysia, South Africa, Mexico and Spain have replicated the TTT course in their countries. This demonstrates not only the capacity of the programme to reach large numbers, but also the sustainability of the programme to deliver future programmes by trained participants themselves in their own countries.

## Supplementary information

**Additional file 1:** Train-the-Trainers in hand hygiene course programme

**Additional file 2:** The World Health Organization's 'My 5 Moments for Hand Hygiene' observation form

**Additional file 3:** Train-the-Trainers in hand hygiene course questionnaire

**Additional file 4: Figure S1.** Train-The-Trainers: Improvement in Hand Hygiene Knowledge. Percentage of correct answers to the pre- and post-course questionnaire section on the knowledge about microbial transmission, HAIs and key principles for hand hygiene best practices **Fig. S2.** Train-The-Trainers: Improvement with Hand Hygiene WHO methodology. Percentage of correct answers to the pre- and post-course questionnaire on the WHO methodology for hand hygiene observations **Fig. S3.** Train-The-Trainers: Improvement in Hand Hygiene Direct Observations. Percentage of correct answers to the pre- and post-course questionnaire on clinical scenario type questions following *My 5 Moments for Hand Hygiene*

## Data Availability

The datasets used and/or analysed during the current study are available from the corresponding author on reasonable request.

## Abbreviations

HAIs: Healthcare-associated infections
HCFs: Healthcare facilities
HCWs: Healthcare workers
HHSAF: Hygiene Self-Assessment Framework
IPC: Infection prevention and control
IPC/WCC: Infection Prevention and Control programme and WHO Collaborating Centre on Patient Safety
LMIC: Lower middle income countries low-and-middle-income countries
MoH: Ministry of Health
TTT: Train the Trainer
WHO: World Health Organization
