# Correction to: Train-the-Trainers in hand hygiene: a standardized approach to guide education in infection prevention and control

**DOI:** 10.1186/s13756-020-0687-z

**Published:** 2020-01-31

**Authors:** Ermira Tartari, Carolina Fankhauser, Sarah Masson-Roy, Hilda Márquez-Villarreal, Inmaculada Fernández Moreno, Ma Luisa Rodriguez Navas, Odet Sarabia, Fernando Bellissimo-Rodrigues, Marcela Hernández-de Mezerville, Yew Fong Lee, Mohammad Hassan Aelami, Shaheen Mehtar, Américo Agostinho, Liberato Camilleri, Benedetta Allegranzi, Daniela Pires, Didier Pittet

**Affiliations:** 1 Infection Control Programme and WHO Collaborating Centre on Patient Safety, University of Geneva Hospitals and Faculty of Medicine, 4 Rue Gabrielle-Perret-Gentil, 1211 Geneva, Switzerland; 2 Institute of Global Health, Faculty of Medicine, University of Geneva, Geneva, Switzerland; 3 Faculty of Health Sciences, University of Malta, Msida, Malta; 4 Hotel-Dieu de Lévis, Lévis, Canada; 5 Department of Public Health, University of Guadalajara, Guadalajara, Jalisco Mexico; 6 Corporación Sanitaria Parc Taulí de Sabadell, Barcelona, Spain; 7 Hospital Universitario Principe de Asturias, Madrid, Spain; 8 Universidad Anáhuac, Naucalpan de Juárez, Mexico; 9 Department of Social Medicine, Ribeirão Preto Medical School, University of São Paulo, Ribeirão Preto, Brazil; 10 Hospital Nacional de Niños, de Costa Rica Dr. Carlos Sáenz Herrera, San José, Costa Rica; 11 Ministry of Health, Riyadh, Saudi Arabia; 12 Department of Pediatrics and Hand Hygiene and Infection Control Research Center, Imam Reza Hospital, Mashhad University of Medical Sciences, Mashhad, Iran; 13 Infection Control Africa Network, Unit of IPC, Tygerberg Hospital, Cape Town, South Africa; 14 Department of Statistics and Operations Research, Faculty of Science, University of Malta, Msida, Malta; 15 Infection Prevention and Control Global Unit, Department of Service Delivery and Safety, World Health Organization, Geneva, Switzerland; 16 Department of Infectious Diseases, Centro Hospitalar Lisboa Norte and Faculdade de Medicina da Universidade de Lisboa, Lisbon, Portugal

**Correction to: Antimicrob Resist Infect Control**

**https://doi.org/10.1186/s13756-019-0666-4**

The original article [1] contained a misspelling in author, Fernando Bellissimo-Rodrigues’s name which has since been corrected.
