# Supplementary material for: Train-the-Trainers in hand hygiene: a standardized approach to guide education in infection prevention and control
Source: Antimicrob Resist Infect Control. 2019 Dec 30;8:206. doi: 10.1186/s13756-019-0666-4 (PMC6937710; doi:10.1186/s13756-019-0666-4)

COURSE FACULTY:

CHAIR

- **Prof Didier Pittet:** Director - Infection Control Programme & WHO Collaborating Centre on Patient Safety, Geneva

COURSE ORGANIZERS AND FACULTY

- **Dr Daniela Pires**: Medical doctor - Infection Control Programme & WHO Collaborating Centre on Patient Safety, Geneva
- **Ermira Tartari:** Infection Control Practitioner and Research Fellow - Infection Control Programme & WHO Collaborating Centre on Patient Safety, Geneva
- **Carolina Fankhauser-Rodriguez:** Microbiologist - Infection Control Programme & WHO Collaborating Centre on Patient Safety, Geneva

FACULTY

- **Americo Agostinho:** Infection control nurse - Infection Control Programme & WHO Collaborating Centre on Patient Safety, Geneva
- **Josiane Sztajzel-Boissard:** Infection control nurse - Infection Control Programme & WHO Collaborating Centre on Patient Safety, Geneva
- **Dr Sarah Masson-Roy**: Medical doctor - Infection Control Programme & WHO Collaborating Centre on Patient Safety, Geneva
- **Dr Fernando Bellissimo Rodrigues:** Medical doctor - Professor of the Social Medicine Department, Ribeirão Preto Medical School, University of São Paulo,
- **Dr Marcela Hernández de Mezerville**: Coordinator, Epidemiology and Infection Prevention and Control Unit - Hospital Nacional de Niños, Costa Rica

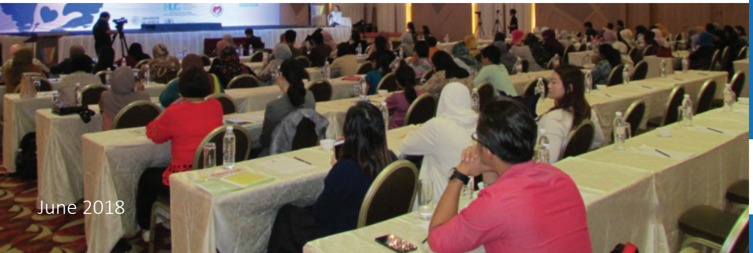

June 2018

COURSE OVERVIEW:

The aim of a Train the Trainers (TTT) course is to set the global standard for training infection prevention and control (IPC) practitioners in hand hygiene.

These key actors establish their institution's hand hygiene infrastructure, lead hand hygiene improvement activities and spearhead scientific research agendas. However, there is widespread variation among the requisite elements of competence in hand hygiene worldwide. The Infection Control Programme and WHO Collaborating Centre on Patient Safety at the University of Geneva Hospitals and Faculty of Medicine, launched a TTT program for advanced and senior IPC practitioners, based on the WHO multimodal improvement strategy, which has been shown to have a measurable positive impact on participants' knowledge. This model sets the stage for standardized training of IPC practitioners worldwide.

The course is organised over 3 days and uses an interactive and multifaceted approach to learning. It uses a combination of group discussion, simulation of clinical scenarios and role-playing with immediate performance feedback. The simulation-based learning in this course allows participants to master the direct observation method for hand hygiene compliance, which is considered the gold standard for evaluation.

Participants' knowledge is assessed through a before-and-after course evaluation tool. This model serves as a reference training method for IPC practitioners and health workers worldwide. Integrate our TTT for hand hygiene into your IPC program to see measurable improvement in your healthcare facility.

More info and videos at: CleanHandsSaveLives.org

TRAIN THE TRAINERS

HAND HYGIENE

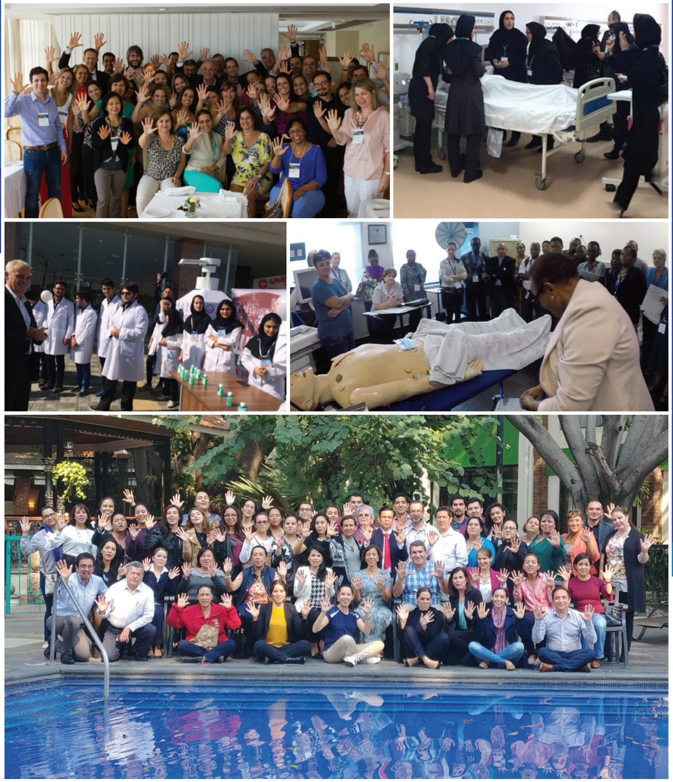

Hôpitaux Universitaires Genève

UNIVERSITÉ DE GENÈVE
FACULTÉ DE MÉDECINE

COURSE PROGRAMME:

DAY 1

09:00 Registration
09:30 Welcome and introduction to the TTT concept
Chair: Professor Didier Pittet
10:00 Pre-course evaluation questionnaire

10:45 COFFEE BREAK

11:15 Global Burden of Health Care-Associated Infections
12:00 The start of Hand Hygiene promotion in Geneva and going Global – the story behind the success

13:00 LUNCH

14:00 WHO Multimodal Hand Hygiene Improvement Strategy
14:45 "My 5 moments for Hand Hygiene" direct monitoring

16:00 COFFEE BREAK

16:30 "My 5 moments for Hand Hygiene" with practical application in clinical scenarios and group discussion (based on audio-visual tools and video reviewing)

18:00 WRAP-UP OF THE DAY

DAY 2

09:00 WHO Hand Hygiene Self- Assessment Framework
How to complete the HHSAF?

11:00 COFFEE BREAK

11:30 How to challenge the campaign fatigue?
Innovations in Hand Hygiene promotion worldwide

12:30 LUNCH

13:30 Simulation based clinical scenarios
Bedside teaching with simulated patients, with role-play performance in groups

15:30 COFFEE BREAK

16:00 Simulation based clinical scenarios
Bedside teaching with simulated patients, with role-play performance in groups

18:00 WRAP-UP OF THE DAY

DAY 3

09:00 What's new in Hand Hygiene?

10:00 Action plan World café - building a hand hygiene action plan: working in groups

11:00 COFFEE BREAK

11:30 Experiences and challenges in Hand Hygiene
Participants' presentations and discussion

13:00 LUNCH

14:30 Post-course evaluation questionnaire

15:30 COFFEE BREAK

16:00 Certificate giving ceremony

17:00 WRAP-UP AND COURSE EVALUATION

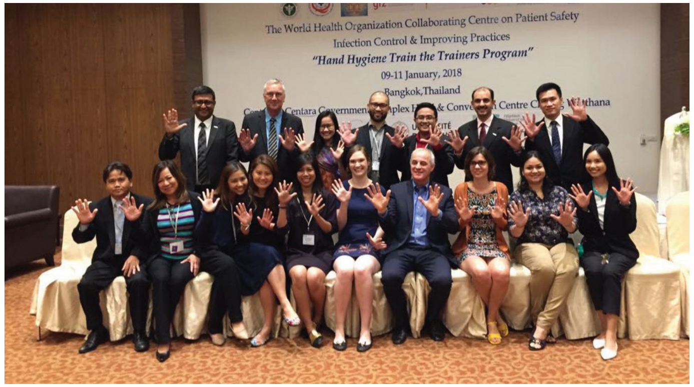

Supplement: Supplementary file 1 — Additional file 1: Train-the-Trainers in hand hygiene course programme [file 13756_2019_666_MOESM1_ESM.pdf]
