# Supplementary material for: Train-the-Trainers in hand hygiene: a standardized approach to guide education in infection prevention and control
Source: Antimicrob Resist Infect Control. 2019 Dec 30;8:206. doi: 10.1186/s13756-019-0666-4 (PMC6937710; doi:10.1186/s13756-019-0666-4)
Supplement: Supplementary file 3 — Additional file 3: Train-the-Trainers in hand hygiene course questionnaire [file 13756_2019_666_MOESM3_ESM.pdf]

Train The Trainers

Hand Hygiene

COURSE QUESTIONNAIRE

Hôpitaux Universitaires Genève
University of Geneva Hospitals and Faculty of Medicine, Geneva, Switzerland

WHO Collaborating Centre on Patient Safety Infection Control & Improving Practices

**Name:**		**Date:**	
**Facility / Hospital name:**		**Years of professional experience:**	
**Position:**		**Years of IPC experience:**	
**City and country:**		**Participant no. *:**	

**** Will be filled by the organisers.***

This questionnaire will assist us in evaluating your knowledge in hand hygiene and competency in hand hygiene observation practices.

**Note: Parts of this questionnaire were adapted from the Hand Hygiene Knowledge Questionnaire for Health-Care Workers*

1. **Regarding healthcare-associated infections (HAIs), which of the following sentences is false (F)? *Select one answer.***

   a) In developed countries, HAIs complicate between 5 to 10% of admissions in acute-care hospitals
   b) In developing countries, this risk of HAI is up to 20 times higher than in developed countries
   c) The definition of HAI does not include infections acquired in the hospital but appearing after discharge
   d) HAI can be caused by microorganisms already present on the patient's skin or mucosa (endogenous microorganisms)
   e) The longer the duration of care, the higher the degree of hand contamination

2. **What is the most frequent source of microorganisms responsible for HAI? *Select one answer.***

   a) The hospital water system
   b) The hospital air
   c) Microorganisms already present on or within the patient
   d) The hospital environment (surfaces)
   e) None of the above

3. **Please state which of the following sentences is false (F)*. Select one answer*.**

   a) Hand hygiene with alcohol-based handrub (ABHR) is more effective in eliminating bacteria than hand washing with soapand water
   b) Even if hands are not visibly soiled, hand washing is preferable to hand rubbing with ABHR, although it takes more time
   c) If exposure to potential spore-forming pathogens such as *Clostridium difficile* is strongly suspected or proven, hand washing with soap and water is preferred
   d) Before handling medication or preparing food, healthcare workers should perform hand hygiene using either ABHR or soap and water
   e) Soap and ABHR should not be used concurrently

**Note: Parts of this questionnaire were adapted from the Hand Hygiene Knowledge Questionnaire for Health-Care Workers*

4. With respect to alcohol-based handrub (ABHR) and handwashing with soap and water: which of the following statements are true (T)?

i) Handrubbing is less time-consuming than handwashing
ii) Handrubbing causes skin dryness more than handwashing
iii) Handrubbing is more effective in eliminating bacteria than handwashing
iv) Handwashing and handrubbing are recommended to be performed in sequence
v) Having long, varnished, or artificial fingernails can increase the chance of microorganism transmission.

***Select one answer. Please* refer to the list above.**

A) i), iii), v)
B) i), iii)
C) iii), iv)
D) iii), iv), v)
E) ii), iii), v)

5. **What are the 5 components of the WHO multimodal strategy for improving hand hygiene? *Select one answer.***

a) Before patient, After patient, Before aseptic, After body fluids, After environment
b) System Change, Training and Education, Evaluation and Feedback, Reminders in the Workplace, Institutional Safety Climate
c) Facility preparedness, Baseline evaluation, Implementation, Follow-up evaluation, Review and planning
d) Hand Hygiene Technical Reference Manual, Hand Hygiene Training Films, Slides for the Hand Hygiene Co-ordinator, Observation tools, Glove Use Information Leaflet
e) Your 5 Moments for Hand Hygiene Poster, How to Handrub Poster, How to Handwash Poster, Hand Hygiene: When and How Leaflet, SAVE LIVES: Clean Your Hands Screensaver

6. Which of the following sentences is false (F)? *Select one answer.*

a) There is an indication for HH whenever there is a risk that hands transmit **microorganisms** during health-care delivery
b) Indications relate to reference points in time i.e. “before” or “after” the contact
c) The five indications designate the moments when hand hygiene is required, in order to effectively interrupt **microorganisms** transmission during care
d) The five moments for hand hygiene are the five "indications" for hand hygiene in healthcare
e) The indications “after patient contact” and “after contact with patient surroundings” can coincide in the same opportunity

7. **What is “an opportunity” for hand hygiene? *Select one answer.***

~~a)~~ An opportunity exists whenever one of the moments for hand hygiene is present
b) All double, triple, or quadruple combined indications may be observed with the exception of “after patient contact” and “after contact with patient surroundings”
c) Each opportunity must be followed by an action (handwashing, handrubbing or missed)
d) Several moments may come together to create a single opportunity
e) All of the above

8. **Regarding the concept of the “patient zone”, which of the following statements is true (T)? *Select one answer.***

a) The patient’s bed frame, bedside table and chair are part of the patient zone.
b) Any dedicated object or surface in the patient's immediate surroundings is part of the patient zone and is considered to be contaminated by the patient's pathogens.
c) The patient zone is the theoretical dotted line which separates the patient environment from the health-care environment
d) For moment 1, you can either:
-First do hand hygiene, then touch the bedside table and the patient
or
-First touch the bedside table, do hand hygiene and then touch the patient
e) All of the above statements are correct

9. **Regarding the moment “before clean/aseptic procedure”, which of the following statements is false (F)? *Select one answer.***

a) Preparation of food, medication and oral/dental care, are examples of care situations where this moment applies
b) This moment includes tasks and procedures which involve contact with the patient's mucous membranes and/or non-intact skin
c) This moment refers to “aseptic” procedures «…(catheter insertion) and also any contact procedure involving contact with mucous membrane/broken skin that constitutes a risk of pathogen inoculation»
d) It is acceptable to skip this moment if I had just performed hand hygiene before touching the patient
e) The term ‘aseptic’ is used in this way in the interests of simplicity, and it is not intended to confuse the standard definitions of what constitutes asepsis

10. **Regarding the use of gloves, please state which of the following sentences are false (F).**

i) The use of gloves replaces the need for hand hygiene
ii) Gloves should be worn when it can be reasonably anticipated that contact with blood or other potentially infectious materials, mucous membranes or non-intact skin will occur
iii) After removing gloves, healthcare workers should handrub or handwash iv) In some situations, you can wear the same pair of gloves for the care of more than one patient
i) When wearing gloves, you should change gloves during patient care if moving from a dirty body site to another body site

***Select one answer. Please* refer to the list above.**

A) i) iii) iv)
B) ii) iv)
C) ii) v)
D) i) iv)
E) none of the above

11. **According to WHO guidelines, up to how many health care workers are you allowed to observe simultaneously during one hand hygiene observation session in general wards? *Select one answer.***

    i. 2
    ii. 3
    iii. 4
    iv. 5
    v. 6

12. **According to WHO guidelines, what is the duration of an observation session? *Select one answer.***

    i. 5 minutes
    ii. 10 minutes (+/- 10 minutes is accepted)
    iii. 15 minutes (+/- 5 minutes is accepted)
    iv. 20 minutes (+/- 10 minutes is accepted)
    v. 30 minutes (+/- 5 minutes is accepted)

13. **According to WHO guidelines, which is *Moment 3* in hand hygiene? *Select one answer.***

    i. After patient surroundings
    ii. After the risk of exposure to body fluid
    iii. After touching a patient

**Note: Parts of this questionnaire were adapted from the Hand Hygiene Knowledge Questionnaire for Health-Care Workers*

iv. Before clean/aseptic procedure
v. Before touching a patient

In the next questions (#14 to 22), you are observing the situations described in the scenarios. Select the completed WHO observation form that best suits the situation described.

Bef-pat.=before patient	HH=Hand Hygiene
Bef-asept. =Before aseptic	HR=Hand rub
Aft-b.f=After body fluids	HW=Hand wash
Aft-pat.=After patient	
Aft-p.surr.=After patient surroundings	

14. A doctor and a nurse enter a room. The doctor shakes hands with the patient while the nurse moves the patient's table to the side. *Select one answer.*

a) 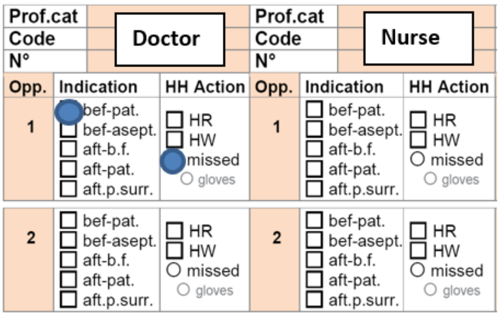

b) 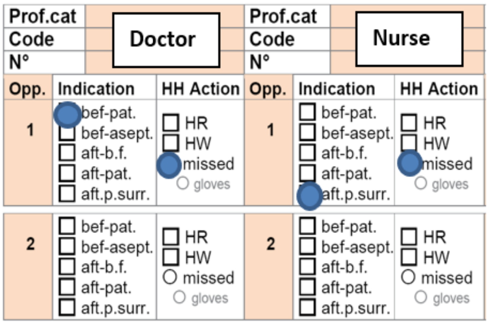

c) 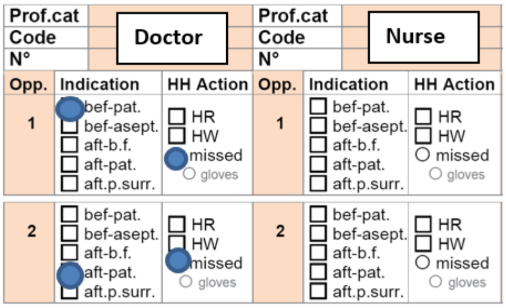

d) 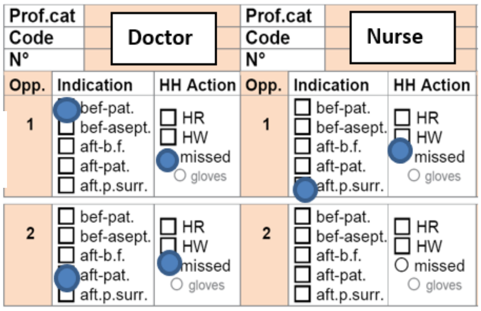

15. A doctor approaches a patient to assess the peripheral venous catheter. During examination, he finds the catheter site warm and the dressing soiled. His phone rings and he answers it. *Select one answer.*

**Note: Parts of this questionnaire were adapted from the Hand Hygiene Knowledge Questionnaire for Health-Care Workers*

a)

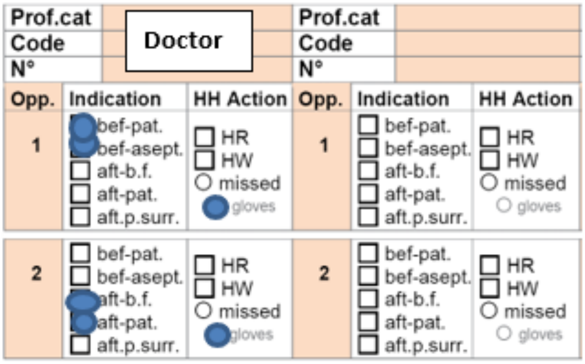

b)

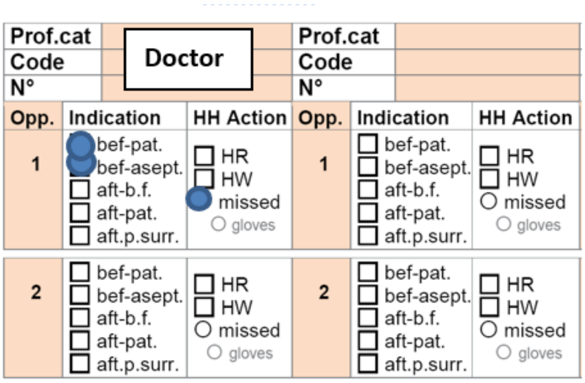

c)

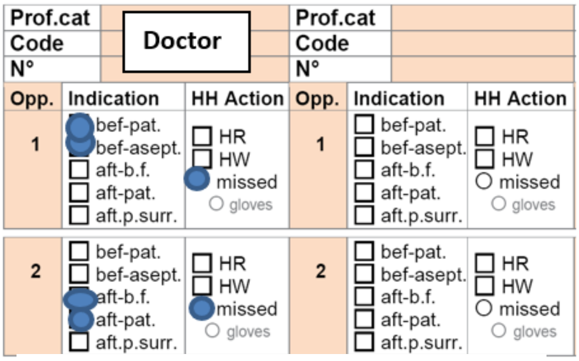

d)

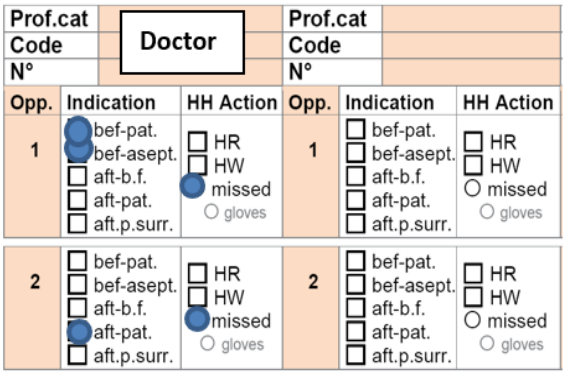

nd ha
atient
get t

a)

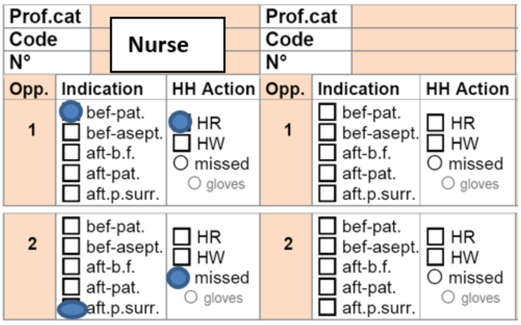

b)

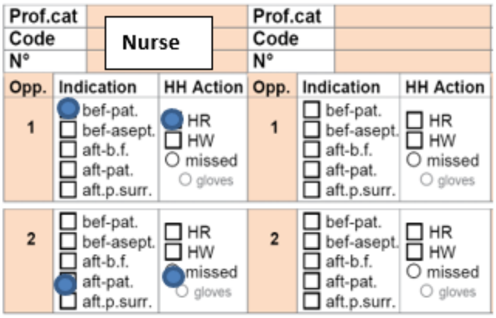

c)

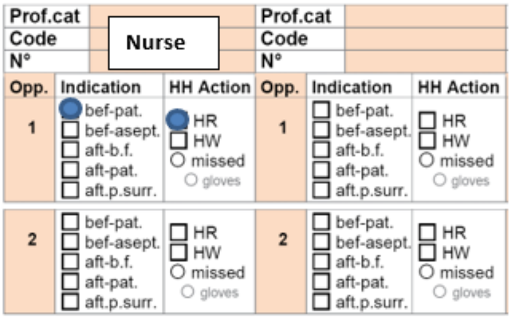

d)

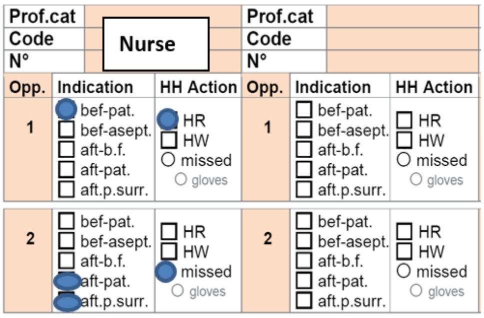

Note: Parts of this questionnaire were adapted from the Hand Hygiene Knowledge Questionnaire for Health-Care Workers

17. **After examining a patient, the doctor leaves the room and handrubs on his way out. At the same time the physiotherapist arrives, handrubs, shakes hands with the patient and they both discuss. *Select one answer.***

a)

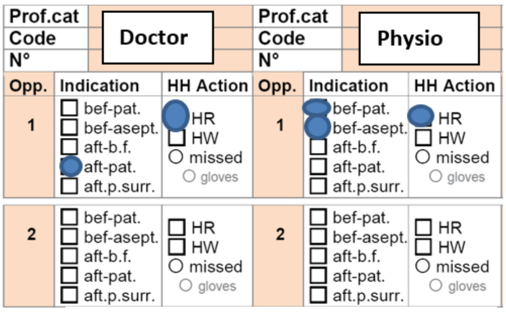

b)

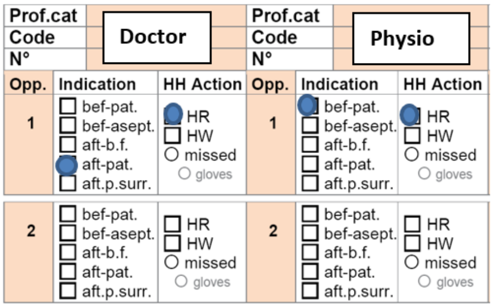

c)

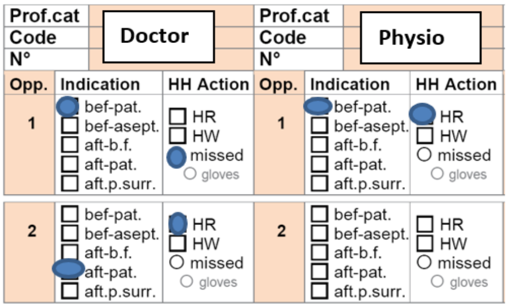

d)

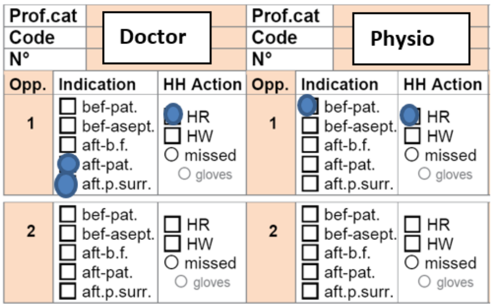

18. **After finishing a physiotherapy session with a patient, the physiotherapist takes the file from the patient's bedside table and leaves the room.**

a)

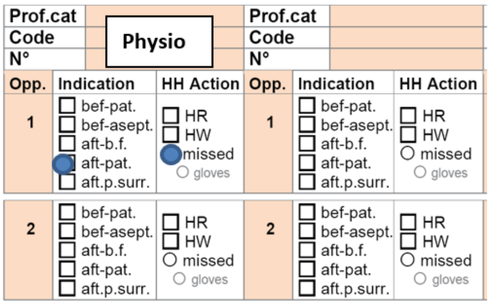

b)

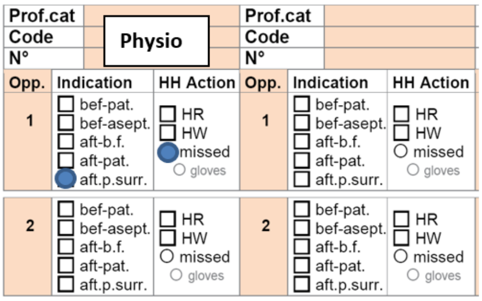

c)

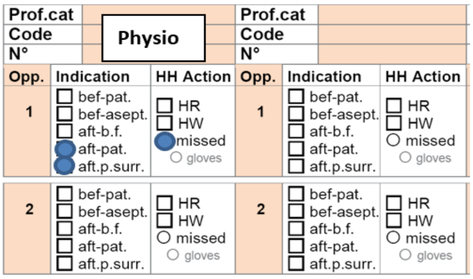

d)

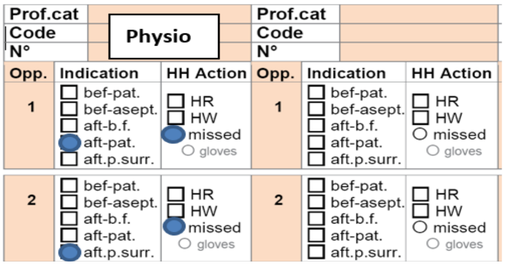

**Note: Parts of this questionnaire were adapted from the Hand Hygiene Knowledge Questionnaire for Health-Care Workers*

19. **A nurse enters a room with the medication trolley. She goes towards the patient, dons gloves and gives an injection. There is blood at the injection site so she uses a swab to stop the bleeding. She then disposes of the material and washes her hands and leaves and the room. *Select one answer.***

a)

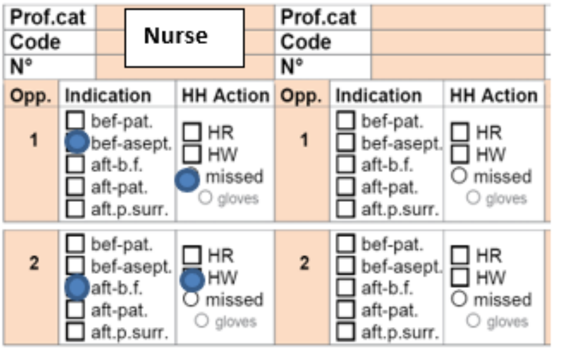

b)

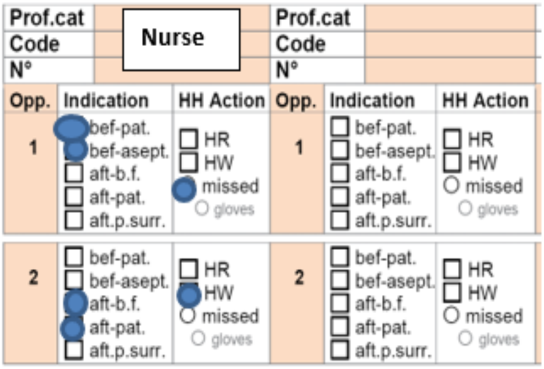

c)

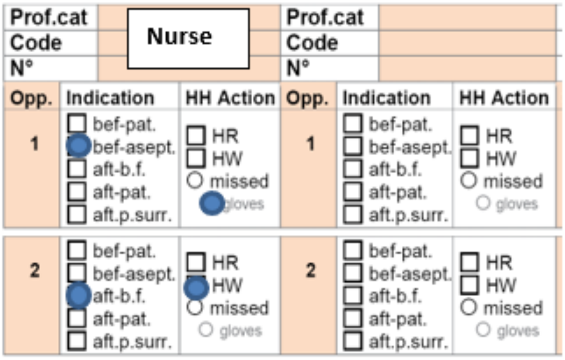

d)

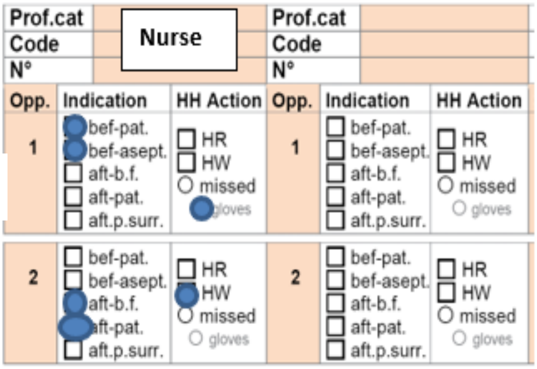

20. **Following removal of the peripheral venous catheter of patient A, the nurse takes off her gloves, goes to patient B and opens the peripheral venous catheter port to give the injection. *Select one answer.***

a)

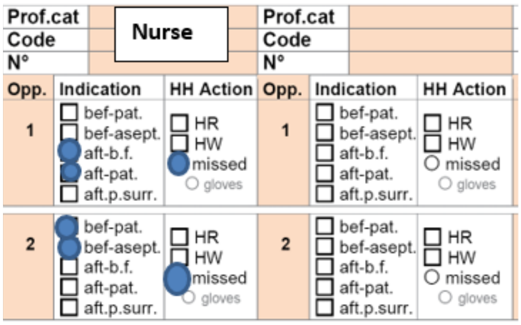

b)

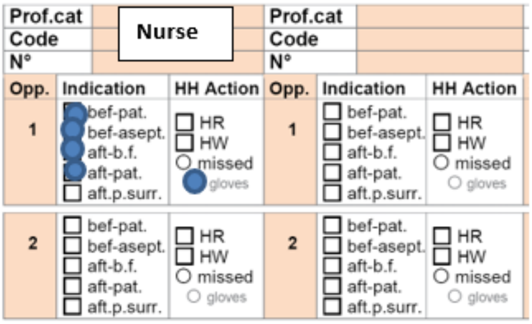

c)

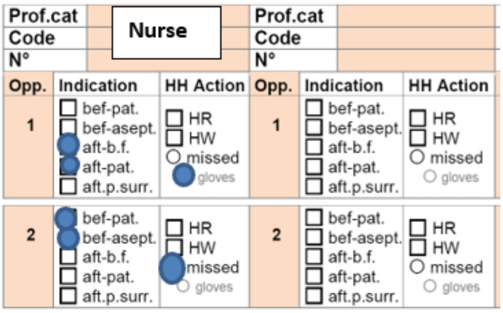

d)

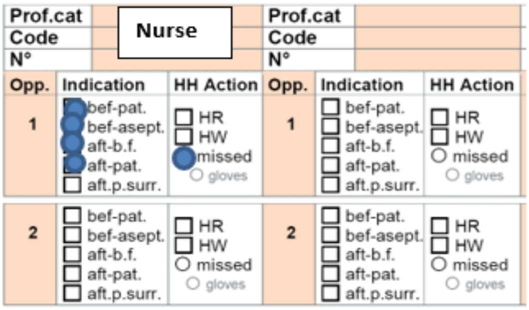

TTT pre

**Note: Parts of this questionnaire were adapted from the Hand Hygiene Knowledge Questionnaire for Health-Care Workers*

21. **A nurse enters a patient's room. She empties the urinary catheter bag. She then washes her hands and leaves the room. *Select one answer.***

a)

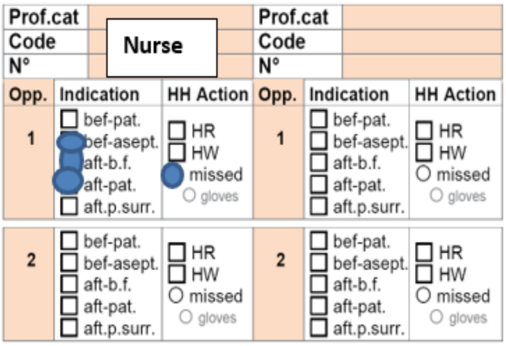

b)

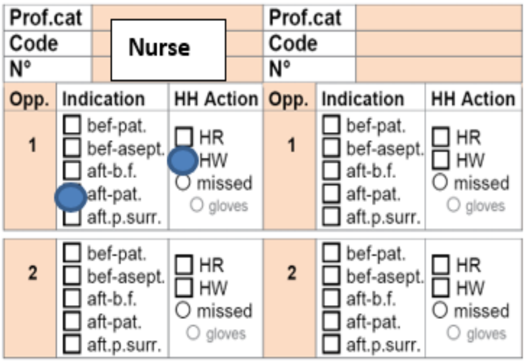

c)

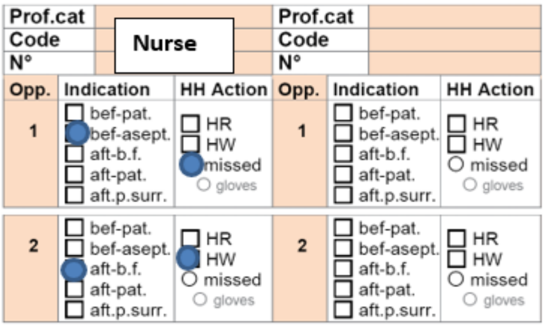

d)

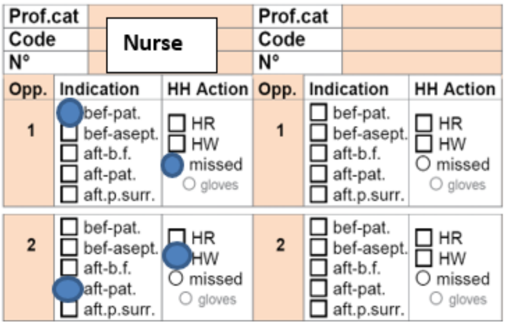

22. **An auxiliary nurse enters a two bedded room and offers patient A a newspaper. She moves the bedside table and places the newspaper on it. She then assists patient B to stand up. The auxiliary nurse handrubs and leaves the room.**

a)

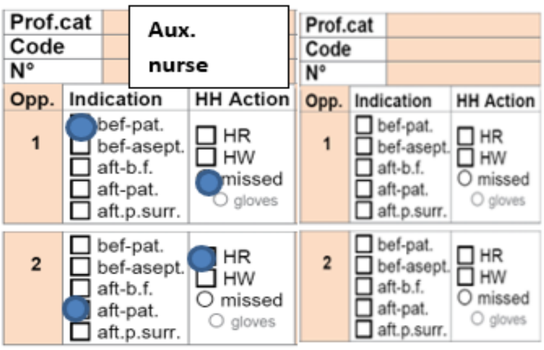

b)

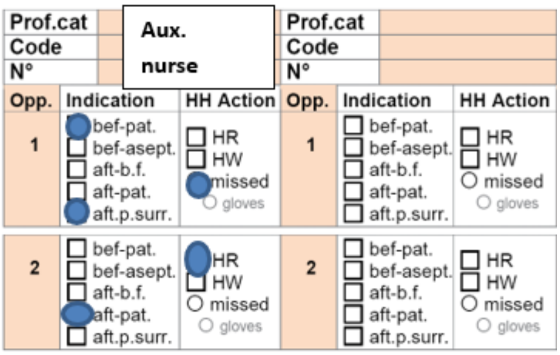

c) 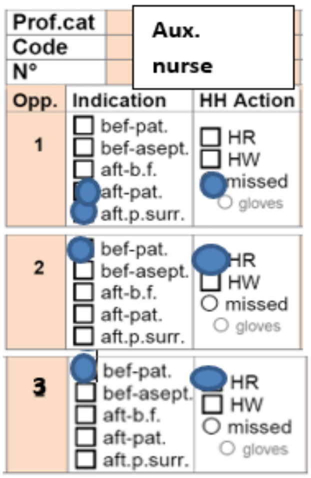

d) 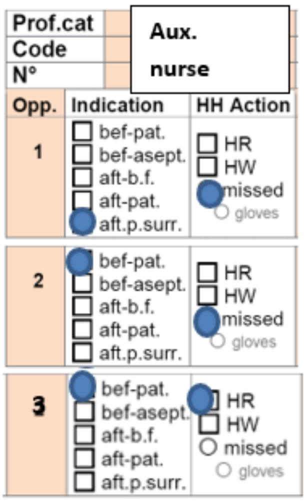

The following section concerns the hand hygiene programme in your healthcare facility.

23. Did your hospital complete the WHO Hand Hygiene Self-Assessment framework?

☐ Yes ☐ No

24. Did your hospital celebrate the last 5th of May "SAVE LIVES Clean Your Hands"?

☐ Yes ☐ No

A. If yes, briefly state what activities you performed:

____

____

25. Does your hospital perform hand hygiene observations regularly? ☐**Yes** ☐**No**

A. If yes, how frequently are hand hygiene observations performed?

- monthly
- quarterly
- biannually
- Annually

B. If yes, which HCWs are observed (select all that apply)?

- Doctors
- Nurses

- Auxiliary staff
- Physiotherapists
- Others, please specify_______________

C. If yes, which departments are monitored (select all that apply)?

- medical
- surgical
- intensive care unit
- rehabilitation wards
- others, please specify ____

26. Does your hospital have a training and validation plan for hand hygiene observers?

☐Yes ☐No

A. If yes, please describe the plan and how it is implemented

____

27. Does your hospital have a training strategy on hand hygiene for HCWs?

☐Yes ☐No

A. If yes, how often HCWs receive hand hygiene training?

- Never
- At least once
- Regular training for medical and nursing staff, or all professional categories (at least annually)
- Mandatory training for all professional categories at commencement of employment, then ongoing regular training (at least annually)

____

B. When was the last time you attended general hand hygiene training?

- Never
- > 5 years ago
- 3-5 years ago
- 1-3 years
- within the last 12 months

C. When was the last time you attended direct observation hand hygiene training?

- Never
- > 5 years ago
- 3-5 years ago

- 1-3 years
- within the last 12 months

<u>THANK YOU FOR COMPLETING THIS QUESTIONNAIRE!</u>

**Note: Parts of this questionnaire were adapted from the Hand Hygiene Knowledge Questionnaire for Health-Care Workers*
