# Supplementary material for: Train-the-Trainers in hand hygiene: a standardized approach to guide education in infection prevention and control
Source: Antimicrob Resist Infect Control. 2019 Dec 30;8:206. doi: 10.1186/s13756-019-0666-4 (PMC6937710; doi:10.1186/s13756-019-0666-4)
Supplement: Supplementary file 4 — Additional file 4: Figure S1. Train-The-Trainers: Improvement in Hand Hygiene Knowledge. Percentage of correct answers to the pre- and post-course questionnaire section on the knowledge about microbial transmission, HAIs and key principles for hand hygiene best practices Fig. S2. Train-The-Trainers: Improvement with Hand Hygiene WHO methodology. Percentage of correct answers to the pre- and post-course questionnaire on the WHO methodology for hand hygiene observations Fig. S3. Train-The-Trainers: Improvement in Hand Hygiene Direct Observations. Percentage of correct answers to the pre- and post-course questionnaire on clinical scenario type questions following My 5 Moments for Hand Hygiene [file 13756_2019_666_MOESM4_ESM.docx]

**Additional File 4**


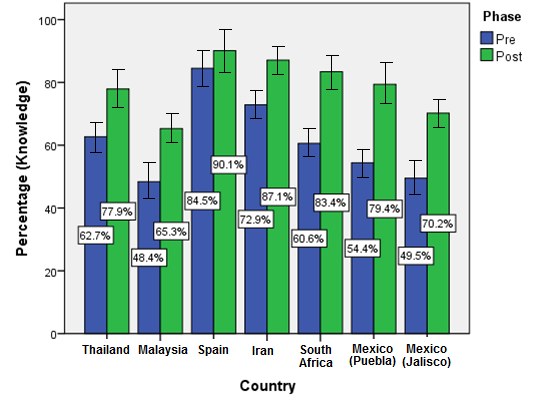


Figure 1 **Train-The-Trainers: Improvement in Hand Hygiene Knowledge.**

Percentage of correct answers to the pre- and post-course questionnaire section

on the knowledge about microbial transmission, HAIs and key principles for hand hygiene best practices


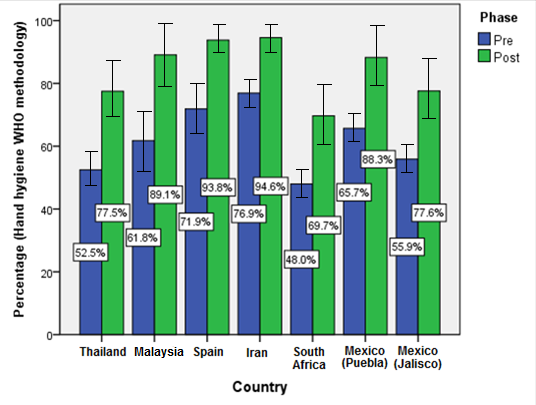


Figure 2 **Train-The-Trainers: Improvement with Hand Hygiene WHO methodology.**

Percentage of correct answers to the pre- and post-course questionnaire on the WHO methodology for hand hygiene observations


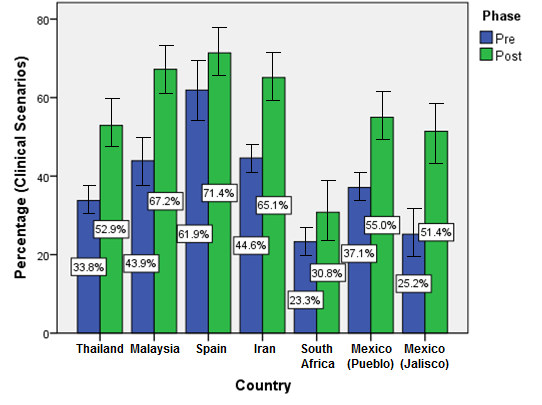


Figure 3 **Train-The-Trainers: Improvement in Hand Hygiene Direct Observations.**

Percentage of correct answers to the pre- and post-course questionnaire on clinical scenario type questions following *My* *5 Moments for Hand Hygiene*
